# Validation of a Persian version of the OIDP index

**DOI:** 10.1186/1472-6831-7-2

**Published:** 2007-01-26

**Authors:** Mojtaba Dorri, Aubrey Sheiham, Georgios Tsakos

**Affiliations:** 1 Department of Epidemiology and Public Health, University College of London, London, UK

## Abstract

**Background:**

Measuring the impacts of oral conditions on quality of life is an important part of oral health needs assessment. For this purpose a variety of oral health-related quality of life instruments have been developed. To use a scale in a new context or with a different groups of people, it is necessary to re-establish its psychometric properties. The objectives of this study are to develop and test the reliability and validity of the Persian version of Oral Impacts on Daily Performances (OIDP) index.

**Methods:**

The Persian version of OIDP index was developed through a linguistic translation exercise. The psychometric properties of the Persian version of OIDP were evaluated in terms of face, content, construct and criterion validity in addition to internal and test-retest reliability. A convenience sample of 285 working adults aged 20–50 living in Mashad was recruited (91% response rate) to evaluate the Persian version.

**Results:**

The Persian version of OIDP had excellent validity and reliability charactersitics. Weighted Kappa was 0.91. Cronbachs alpha coefficient was 0.79. The index showed significant associations with self-rated oral and general health status, as well as perceived dental treatment needs, satisfaction with mouth and prevalence of pain in mouth (P < 0.001). 64.9% of subjects had an oral impact on their daily performances. The most prevalent performance affected was eating, followed by major work or role and sleeping.

**Conclusion:**

The Persian version of OIDP index is a valid and reliable measure for use in 20 to 50 year old working Iranians.

## Background

Measuring the impact of oral conditions on quality of life is an important part of assessing oral health. It is now recognised that there are serious limitations in solely using the clinical normative assessments for the measurement of oral health status and needs. Clinical measures do not consider the individual's perceived health status or perceived needs [1]. Health is no longer defined in terms of illness and disease, but concepts have been broadened to take into account physical, psychological and social aspects of well-being [2]. Hence, measures of health status, which more accurately reflect its multi-dimensional character, have been advocated [3]. These measures, which assess "the extent to which oral conditions disrupt normal social role functioning and lead to major changes in behaviour", are known as socio-dental indicators or oral health-related quality of life measures (OHRQoL) [4-7].

A variety of oral health-related quality of life instruments have been developed in the past 20 years. The Oral Impacts on Daily Performances (OIDP) measure is a commonly used OHRQoL indicator. OIDP is a relatively brief and theoretically sound instrument. It focuses on the assessment of the impacts caused by oral conditions on the person's abilities to perform activities and behaviours of daily life [8].

The OIDP has been used in different studies of adult populations in Great Britain and Greece [9,10], Thailand [11], Tanzania [12], Uganda [13], and Norway [14]. The measure has proved to be reliable and valid in cross-sectional population-based studies as well as in studies of patients with specific oral disorders, such as traumatic injuries and malocclusion [15,16].

Every time a scale is used in a new context or with a different group of people, it is necessary to re-establish its psychometric properties [17]. The objective of this study was to adapt the OIDP index into Persian, the official language of Iran, and to test its reliability and validity in an adult working Iranian sample

## Methods

The process of adapting the OIDP index for adults into Persian and evaluating of its psychometric properties involved three main steps; linguistic translation of the original OIDP into Persian; pilot study to assess face and content validity; and the main study for validity and reliability testing.

Linguistic translation of the original OIDP: The procedure used in this study was mainly based on the method of linguistic validation described by Acquadro et al. [18], with small modifications. We have used the modified version of OIDP developed by Tsakos et al. (2001) [10]. This modified version had been previously used for elderly people in the oral health survey of the British National Diet and Nutrition Survey (NDNS). The item 'carrying out major work or social role' was included in the original version for adults developed by Adulyanon and Sheiham (1997) but not in the modified version, as the relevant question was found irrelevant to the elderly population, since their vast majority consisted of pensioners that did not work. However, as our study refers to adults of working age, the item on 'carrying out major work or social role' was also included here. In the first step, the OIDP index was independently translated into Persian, by two qualified English-to-Persian translators. After a group discussion with the translators and one author (MD), the first consensus Persian OIDP was backward translated to English. The backward translation was compared with the original index and the first consensus Persian OIDP. There were very few differences and did not affect the construct of instrument. For example, the word "denture" in the original copy was translated as "false teeth" in the backward translation. Appropriate changes, mainly in wording, were introduced and therefore, the second Persian version of OIDP was created, which was then pilot tested. Overall, the evaluation showed that the questions in the Persian and English versions were comparable.

### Pilot study

The Persian version of the OIDP was pilot tested to assess its face and content validity in an Iranian population. A convenience sample of 48 working adult Iranians, aged 20–50 -years, all native Persian speakers, resident in Mashad in the far northern east of Iran, were recruited. All had a questionnaire-led interview by one trained and experienced interviewer. The interviewer recorded any difficulty that subjects had encountered and also their comments. All records were reviewed by one of the authors (MD) and a discussion session with the interviewer and some of the subjects was arranged in order to clarify their comments. All necessary changes were made before the main study, including a minor grammatical change to make it more understandable. In addition we relocated the option "tooth pain" in the list of conditions from first to last, for subjects who could not detect a particular cause for their pain.

### Main study

The Persian OIDP was applied to an Iranian population in order to assess its validity and reliability. For this purpose, a sample size of 100–200 is recommended [19]. According to the estimated prevalence of oral impacts in the pilot study (87.5%), and assuming a standard error of 2%, the minimum sample size was 273 people. In order to allow for non-response (estimated to be 10%), at least 300 people should be invited. 312 subjects, working adults aged 20–50 years, all native Persian speakers, living in Mashad, Iran, who were visiting the Shrine Imam Reza in Mashad were recruited. This shrine is visited daily by many people from a variety of social classes. Visitors were approached at the entrance of the shrine and invited to participate in this study.

Ethical approval on human research was obtained from the Iranian National Ethical Committee. All interviewees were briefed about the purpose and process of the study and consent was sought for questionnaire-led interviews and simple oral examination with a mouth mirror.

Each subject was asked about his/her age, sex, occupation and place of residence (urban/peri-urban). Subjects answered the Persian version of the OIDP questionnaire in face-to-face interviews conducted by one trained interviewer. The OIDP questionnaire asks about the oral impacts in relation to major daily performances; eating, speaking, cleaning teeth/dentures, doing light physical activities, going out, sleeping, relaxing, smiling, emotional stability, enjoying the contact of other people and carrying out main role or work. For each reported oral impact, its frequency and severity were further assessed. Finally, each impact was attributed to specific oral conditions, as indicated by the respondents. The OIDP score is expressed as the sum of the different Performance scores (Performance score = severity score × frequency score) divided by the maximum possible score, and then multiplied by 100 to provide a percentage score.

As there is no universally accepted "gold standard" indicator [20] to test the OIDP index against, the single-item assessment of perceived treatment need was used as a proxy because one key property of the index is to contribute to needs assessment. In cases where there is no clear "gold standard", the role of construct, rather than criterion, validity becomes more crucial. Apart from perceived treatment need, subjects were asked about their perceived general health, oral health as well as oral health in relation to general health, satisfaction with mouth and pain in mouth in the past 6 months. These questions were included to assess the construct validity of instrument. It was hypothesised that subjects with higher OIDP scores would report higher self-rated treatment need and pain in the last six months, and have worse self-rated oral and general health. They would also be less satisfied with their mouth and rate their oral health lower than their general health.

Finally, to assess the wording and structure of questionnaire, subjects were asked about the difficulty they had understanding the questions and completing the interview.

### Data analysis

The analysis of the study was carried out using the Statistical Package for Social Sciences (SPSS). The cut-off level for statistical significance was taken at 0.05. The internal consistency of the Persian OIDP was assessed by standardised Cronbach's alpha, alpha if item deleted, inter-item, and item-total correlation coefficients. Test-retest reliability was assessed by the weighted kappa, using the data from 40 subjects who were re-interviewed two weeks after the first visit. As the OIDP scores were not normally distributed, testing for criterion and construct validity was carried out using non-parametric tests; Mann-Whitney and Kruskal-Wallis, as applicable.

## Results

Three hundred and twelve working adults living in Mashad were invited to participate in the study. 285 answered the interviewer-administered questionnaire; a response rate of 91%. The majority of the sample were male (56.8%). The mean age was 36.9 ± 7.7 years. The frequency distribution by occupation groups is shown in Table 1. Only 38.6% rated their oral health as "good" or "very good". 37.6% were "considerably" or "a great deal" satisfied with their mouth and 19% perceived no need for dental treatment. 29% of respondents perceived they had no dental pain in the last 6 months (Table 2).

**Table 1:** Socio-demographic profile of the sample (N = 285)

Variables	Percent
**Age groups**	
20–25 years	7.7
26–30	16.1
31–35	17.9
36–40	17.9
41–45	23.9
46–50	16.5
**Sex**	
Male	56.8
Female	43.2
**Occupations (groups)**	
Upper managerial staff, professionals	9.1
Intermediary occupations	21.4
Craft, trade and firm managers	11.6
Clerks and trade related employees	25.6
Skilled manual workers	19.6
Unskilled manual workers	17.9

**Table 2:** Subjective measures of oral and general health (N = 285)

Variables	Percent
**Perceived general health status**	
Very poor	0.0
Poor	2.5
Fair	24.6
Good	42.5
Very good	30.5
	
**Perceived oral health status**	
Very poor	5.3
Poor	21.4
Fair	34.7
Good	30.5
Very good	8.1
	
**Comparison of Perceived oral health to general health.**	
Superior	11.9
Equal	52.3
Inferior	21.1
Not comparable	14.7
	
**Perceived satisfaction with mouth**	
Not at all	8.8
Very little	22.1
To some extent	31.6
Considerably	32.3
A great deal	5.3
	
**Perceived need for dental treatment**	
Not at all	18.9
Very little	24.2
To some extent	31.6
Considerably	21.4
A great deal	3.9
	
**Perceived dental pain in mouth in past 6 months**	
No	29.1
Yes, not severe	46.0
Yes, severe	14.0
Yes, very severe	10.9

The prevalence of oral impacts, measured by the Persian OIDP index, was very high. 64.9% of participants experienced at least one OIDP impact in the last 6 months (Table 3). The mean OIDP score for the population was 4.15 ± 5.94. The most prevalent OIDP impact was "difficulty eating", reported by 35.1% of respondents. A variety of other oral impacts were also prevalent; "difficulty carrying out main role or work" was reported by 22.1% and "difficulty sleeping" by 21.8%. Relatively fewer, but still considerable proportion of respondents experienced difficulties in "showing teeth while smiling", "enjoying the contact of other people" and "relaxing"; 18.2%, 17.5% and 14.4% respectively. The remaining impacts were less prevalent. The least affected daily performance was "going out" (4.2%). The impacts on "eating" had a high frequency. The same was not the case about severity. 17.2% of the sample experienced an eating impact "nearly every day" or for a spell of "more than three months", but only 0.6% reported that it had a "severe effect" on their daily life while 17.2% said it had "no effect".

**Table 3:** Prevalence of oral impacts on daily performances (N = 285)

Performance	Percent
Eating food	35.1
Speaking	11.6
Cleaning teeth or dentures	10.2
Doing light physical activities	7.7
Going out	4.2
Sleeping	21.8
Relaxing	14.4
Smiling, laughing without embarrassment	18.2
Emotional state; becoming easily upset	9.5
Enjoying the contact of other people	17.5
Carrying out main role or work	22.1
Any impact	64.9

Face and content validity were established in the pilot study and also evaluated by including two questions in the main study. More than 90% of subjects did not find the OIDP difficult to understand and reported it as "somewhat easy" (55.1%) or "very easy" (37.5%). When asked about the difficulty of answering the interviewer-administered questionnaire, almost 90% of subjects said it was somewhat easy (59.3%) or very easy (29.5%).

In terms of internal reliability, the standardized Cronbach's alpha for the OIDP was 0.79. The alpha coefficient did not increase when any of the items were deleted. The inter-item correlations among the OIDP items were mostly positive, with the highest belonging to the relationship between "sleeping" and "relaxing" (0.83). Only four of the correlations were negative. The smallest was the correlation between "smiling" and "eating" (-0.09). The corrected item-total correlations ranged from 0.069 for the item "speaking" and 0.098 for "smiling", to 0.674 for the question on "sleeping" (Table 4). In terms of test-retest reliability, the weighted kappa statistic was 0.91.

**Table 4:** Reliability Analysis using Cronbach's alpha

	Corrected Item-total Correlation	Cronbach's Alpha if Item Deleted
Eating	.579	.71
Speaking	.069	.77
Cleaning teeth/denture	.422	.73
Doing light physical activities	.526	.73
Going out	.445	.74
Sleeping	.674	.70
Relaxing	.655	.71
Smiling	.098	.78
Emotional stability	.567	.73
Social contact	.225	.77
Carrying out main role	.597	.71

Standardised item alpha = 0.79

Regarding criterion validity, the results indicated that adults who perceived a need for dental treatment had much higher OIDP scores than those who did not perceive dental treatment need (p < 0.001) (Table 5). For construct validity, there was a highly significant relationship (p < 0.001) between OIDP and perceived general health, oral health, oral health in relation to general health, satisfaction with mouth and pain in mouth in the past 6 months. Those who perceived their oral health or general health to be worse were more likely to have a higher OIDP score. People who ranked their oral health higher compared to their general health were less likely to have oral impacts on their quality of life. In addition, the association between OIDP scores and perceived satisfaction with mouth revealed that those with higher OIDP scores had lower prevalence of satisfaction with their oral health. Furthermore, subjects with toothache in the past 6 months had higher OIDP scores than those without toothache, and there were also clear differences in the prevalence of oral impacts according to the grade of the pain experience.

**Table 5:** Construct and criterion validity. Comparison of OIDP scores and self-rated measures of oral health and needs (n = 285)

	Mean (SD)	Quartiles	P value
**Perceived dental treatment need**			
Not at all	0.7 (2.0)	(0.0, 0.0, 0.0)	< 0.001
Very little	1.3 (1.6)	(0.0, 0.0, 2.0)	
To some extent	4.6 (4.8)	(1.5, 3.3, 6.5)	
Considerably and a great deal	8.9 (8.1)	(3.7, 6.9, 10.2)	
**Perceived oral health**			
Very poor	9.6 (6.3)	(4.4, 9.5, 14.2)	< 0.001
Poor	8.9 (8.2)	(4.0, 6.6, 9.8)	
Fair	3.4 (4.4)	(0.0, 2.1, 4.7)	
Good	1.7 (2.6)	(0.0, 0.0, 1.8)	
Very good	0.0 (0.0)	(0.0, 0.0, 0.0)	
**Perceived oral health vs. general health**			
Superior	1.4 (2.9)	(0.0, 0.0, 1.8)	< 0.001
Equal	3.2 (5.6)	(0.0, 1.8, 4.4)	
Inferior	7.9 (7.7)	(1.8, 6.6, 11.2)	
**Perceived general health**			
Poor and Fair	7.3 (7.4)	(1.3, 4.4, 10.9)	<0.001
Good	3.7 (5.7)	(0.0, 2.2, 5.3)	
Very good	2.0 (3.1)	(0.0, 0.0, 3.6)	
**Satisfaction with mouth/teeth**			
Not at all	8.5 (8.3)	(4.0, 6.5, 9.1)	< 0.001
Very little and To some extent	5.3 (6.1)	(1.5, 4.1, 7.9)	
Considerably	1.1 (2.1)	(0.0, 0.0, 1.8)	
A great deal	0.7 (2.0)	(0.0, 0.0, 0.0)	
**Prevalence of pain in mouth**			
No	1.5 (3.0)	(0.0, 0.0, 1.8)	< 0.001
Yes, not severe	3.2 (3.8)	(0.0, 1.8, 5.1)	
Yes, severe	7.7 (8.3)	(2.6, 4.5, 8.7)	
Yes, very severe	10.7 (8.6)	(5.5, 9.8, 13.1)	

For the aforementioned relationships, the results showed a clear trend throughout all categories and not only for differences between subjects in the extremes of the OIDP scores distribution (Table 5).

## Discussion

An instrument adapted for use in another country or culture should be culturally relevant and valid for the local population, while demonstrating acceptable psychometric properties. Thus, it is important to carry out a rigorous translation and validation process before an instrument developed in one culture is used in a different cultural setting [14,21-23]. This study was the first to use the OIDP index on an Iranian population. It was also the first study of oral health-related quality of life measurement applied in this age group in Iran. There was only one generic health-related quality of life index, namely the SF-36, validated and adapted for this population in Iran [22].

The Persian version of OIDP was reliable and valid for use among 20–50 year old working adults in Iran. Face and content validity were established in the pilot study and confirmed in the main study. Construct validity was assessed by investigating the relationship between the Persian OIDP index and subjective oral and general health measures. The results showed significant associations among subjective health status and OIDP scores (p < 0.001). This suggests that those subjects who perceived higher dental treatment need were more likely to have an impact on their quality of life. The same applies to other questions; for example, those who were more satisfied with their mouth or perceived less pain in their mouth were less likely to have impacts on their quality of life. This suggests excellent criterion and construct validity for the Persian OIDP. Clinical measures were not considered in the validity testing, since numerous studies have identified a difference between professionally and self-defined oral health, stemming from the conceptual distinction between health and disease [24]. Studies suggest that professional and lay people's oral health concepts differ in that they measure different dimensions of human experience, which are conceptually and often empirically distinct and have different implication for treatment [25-27].

The internal reliability was successfully tested in various ways [17]. All corrected item-total correlations were above the minimum recommended level of 0.20 [28] for being included in a scale, with the exception of those for "speaking" and "smiling". Nearly all inter-item correlations were positive and no correlation was high enough for any item to be redundant. A few inter-item correlations were negative, but still very close to zero. Cronbach alpha coefficient was 0.79. Although there is no actual lower limit to the coefficient [29], this value is higher than the recommended value of 0.70 [30]. This is further evidence to show the results corresponded to previous studies on the OIDP index [10,14,23,31]. The results of the analyses in this study showed very good internal reliability and demonstrated the homogeneity of items. Furthermore, the results of the test-retest procedure provided adequate evidence in relation to the external reliability of the index. The weighted kappa (0.91) was above the recommended threshold of 0.75 [32].

The prevalence of oral impacts on daily performances was 64.9%. This is consistent with the subject's perceived oral health status, as less than 40% of subjects perceived their oral health "good" or "very good". Population-based studies in UK reported lower values for oral impacts for UK adult populations [33,34]. However, considering that in these studies different OHRQoL measures (OHIP-14 and OHQoL-UK respectively) were used, their direct comparability with our study is somehow limited. Nuttall et al. [33] showed that 51% of dentate adults in UK had their lives affected in some way by their oral status. The prevalence was even lower in a national sample of free-living elderly people (older than 65) in the UK, using the same index [9]. In Thailand, 35–44-year-old subjects had an even higher (73.6%) prevalence of oral impacts than in the UK [11]. The most commonly affected daily performance was "eating", a common finding in other populations using OIDP [8,13,15,23,35].

## Conclusion

In conclusion, the Persian version of the OIDP index is valid and reliable for use in 20–50 year old working adult Iranians. The index can be applied in a larger study in Iran to assess oral health related quality of life.

## Competing interests

The author(s) declare that they have no competing interests.

## Authors' contributions

AS and GT made substantial contributions to conception and design, data analysis and interpretation, and also to revising the manuscript. MD carried out the study and drafted the manuscript. All authors read and approved the final manuscript.

## Pre-publication history

The pre-publication history for this paper can be accessed here:


